# Why did they get in trouble? The influence of firm characteristics and institutional distance on Chinese firms’ foreign market entry attempt

**DOI:** 10.3389/fpsyg.2022.972384

**Published:** 2022-09-06

**Authors:** Shuo Zhang

**Affiliations:** School of Business, China University of Political Science and Law, Beijing, China

**Keywords:** Chinese OFDI, market entry attempt, firm characteristics, institutional distance, emerging market

## Abstract

Despite the rich body of research on the outward foreign direct investment (OFDI) by Chinese multinationals, little attention has been given to the fact that China’s OFDI is facing a high failure rate even in their initial attempt to enter a foreign market. Grounded on institutional theory, this study provides a nuanced view of the expansion dynamic of Chinese multinational firms overseas using a unique dataset that contains both successful and troubled Chinese foreign market entry attempts between 2018 and 2021. We find that at the firm level, state-owned firms are more likely to face difficulties when trying to enter a new market compared to their private counterparts. Firms’ corporate social responsibility (CSR) reporting reduces the chance of failure. These effects are conditioned on the political, economic, and cultural distances between the home and host counties.

## Introduction

Accompanied by the rise of emerging economies, firms from those economies are gaining attention from international business (IB) scholars. Emerging market multinational enterprises (EMNEs) follow the rules of the global market and share some of the characteristics of their western counterparts. However, because of their comparatively short history of internationalization and unique domestic institutional settings, some EMMEs exhibit distinct internationalization dynamics. Compared to their western counterparts, they are confronted with different difficulties associated with extra costs when trying to do business abroad (Cao and Alon, 2021).

Buckley et al. (2007a), the IB literature has swelled with articles regarding EMNEs’ international expansion including determinants of entry mode choices (Surdu et al., 2018), marketing strategies (Rana et al., 2020), and corporate social responsibility (CSR) initiatives (Park, 2018; Tashman et al., 2019). An internationalization process entails risks. In terms of the process of international investment, the target of extant studies are firms that were successful in their initial attempt at international investment. For example, publicly listed firms’ international expansion and merge and acquisition transactions are frequently used by IB scholars. Despite their subsequent performance, these transactions were successful in terms of making the internationalization movement. An attempt at foreign market entry comes with risk (Eriksson et al., 2015) and does not necessarily guarantee that a firm can successfully move to the next step for a foreign market entry attempt. Little is known about the steps before the actual internationalization. This gave rise to the questions such as what happened to those offers and negotiations that did not lead to an actual transaction and what are the influencing factors?

Like their western counterparts, EMNEs suffer from liability of foreignness (LOF) when operating in a distant market (Gaur et al., 2011; Zeng and Xu, 2020). Costs stem from unfamiliarity, contradictory rationale and discriminatory hazards often persist long, and put foreign firms in a disadvantageous position as compared to domestic firms (Eden and Miller, 2004). One source of LOF derives from firm-specific characteristics. Existing studies have documented how firms overcome LOF through possession of ownership-specific advantages (Eden and Miller, 2004; Gaur et al., 2011), such as financial and managerial resources and intangible assets (Nachum, 2003). Many EMNEs do not possess such resources and their pursuit of internationalization follows quite different trajectories than multinationals from developed economies. This resulted in their lack of legitimacy when trying to enter a new market. Doubts and questions arise alongside their rapid internationalization.

This situation is further intensified by the gap between home and host institutional settings. The macro institutional level is beyond the control of any individual firm and is moderating firms’ attempts and efforts. There are ways in which firms can better equip themselves to deal with this situation, such as hiring a CEO with overseas experience or conforming to international practices and standards like certification of ISO quality systems. However, the home country institutions in which the firm is embedded and the host country institutions that firms encounter are sometimes of greater importance in determining firm-level attempts and outcomes. In a narrow sense, a successful foreign market entry attempt through investment means surviving and thriving in the subsequent operation in the foreign market. However, market entry is a complicated and multi-phase process that involves at least two steps: entry and survival. Before having the opportunity to consider how to blend into the local market, first and foremost, a firm has to show the local government, community, and/or partners its capabilities and eligibilities to successfully get permission to do business there (Cao and Alon, 2021). No future steps will be possible without this initial success.

China’s experience is a good representation of the integrating process of burgeoning emerging economies. China is now ahead of Japan and has become the second largest economy (World Bank, 2018^1^). Although China only ranks 84th in GDP per capita, it had a total foreign currency reserve of 3.2 trillion dollars as of 2021 and the amount continues to soar to greater heights as the country recoveries from the COVID-19 pandemic. However, besides the astonishing accumulative OFDI amount and rapid international expansion of Chinese firms, the number of troubling cases reported is also increasing. One most recent example is China Communications Construction Company (CCCC), which was removed from Mexico’s 10 billion Tren Maya Rail Project in June 2022. CCCC was confirmed to construct section “Results and findings” of the railway project that connects the tourist hotspots of Cancun and Tulum before the Mexican government called a halt on the project. The reason for the cancelation was reported to be associated with potential “political risk” and “environmental concerns.”

This study provides a nuanced view of Chinese firms’ internationalization by focusing on the initial step of such a process. Internationalization for firms is a gradual process that involves several steps. Market entry attempt deserves more attention since it is a key initial step for firms’ international expansion. In this study, we extend existing research on Chinese OFDI and focus not only on the successful Chinese foreign investment cases but also on the ones which were not successful in their initial attempts in entering a foreign market. To provide empirical support for our research, we construct a unique dataset that contains both successful and failed/troubled OFDI projects by Chinese firms. Our empirical results indicate that micro-level firm characteristics, such as CEO background and firm overseas experience, and macro-level institutional distance, including the political, economic, and cultural distance between China and host countries, determine Chinese firms’ foreign market entry attempts. In addition, we argue that the extent to which firm-level characteristics affect the outcome of market entry is moderated by macro-level institutional difference—in other words: institutional distance.

## Outward foreign direct investment and troubled market entry attempts: The case of China

As one of the most vibrant economies in the world, China provides a perfect case to study how domestic institutions and IB institutions shape business outcomes. China’s development path has been widely seen as unique as it transitions from a centrally planned economy to a market economy with gradual privatization and marketization, massive private capital inflows, and extensive exporting. Beginning with exporting (Rehman and Ding, 2020; Rehman et al., 2020), China has accumulated ample foreign currency reserve. With the development of domestic institutional settings and infrastructure (Rehman and Noman, 2020, 2021), Chinese firms are increasingly active in the international arena. In recent years, the outflow of Chinese capital has increased considerably. China became the largest developing country investor in 2019 (UNCTAD, 2020^2^). According to a report released by China’s Ministry of Commerce, the National Bureau of Statistics, and the State Administration of Foreign Exchange, China’s OFDI in 2020 hit $153.71 billion, ranking first place globally for the first time ever.^3^

A growing number of Chinese multinationals are engaging in outward foreign direct investment (OFDI) that was commonly considered the privilege of firms from economies at more advanced stages. China surpassed Japan in 2013 and has become the second largest source of capital outflow in the world (UNCTAD, 2013). While large state-owned enterprises (SOEs) remain the main players, private enterprises (PEs) are quickly catching up, and according to statistics published by the Department of Commerce, by the end of 2020, PEs contributed 54.3% of China’s non-financial OFDI. In this process, as Chinese firms keep accumulating experience and confidence, there are remarkable success stories outside the “politically important” fields of natural resources and energy. Recent examples include Shandong Ruyi’s purchase of a controlling stake in Bally from JAB Holding in 20, expanding to the fashion industry, and Venus Medtech, a leading innovative Chinese structural heart disease treatment company, announced its acquisition of 100% equity interest in Cardiovalve Ltd., a pioneering transcatheter mitral and tricuspid valve treatment company.

Despite the encouraging amount of capital outflow, there is a phenomenon that should not be neglected: Chinese OFDI is facing a high failure rate. In a narrow sense, failure means the debacle of a project/investment or simply that a project/investment is not making the profit as it was supposed to. According to Sina Finance—a leading Chinese financial media company—about 70% of Chinese overseas projects are losing money and about half of existing projects will probably fall apart in the coming decade.^4^ In a broader sense, failure does not necessarily mean being unsuccessful after the investment was made or the agreement sealed; it also indicates failure of the attempt to enter the foreign market in the first place, which has not been paid the amount of attention it should have.

In fact, more than half of high-profile M&A deals announced by Chinese MNEs have been abandoned (Sun et al., 2012) and little is known about the causes and reasons for those incomplete acquisitions (Peng, 2012). Existing Chinese OFDI research employing institutional theory has focused mainly on firms that have passed the initial entry phase and are at their survival (Peng et al., 2008; Sun et al., 2012) and cases, where firms failed at the initial entry phase, have been largely ignored (Peng, 2012). Therefore, probing into the question of how macro-level institutional settings moderate firm-level attempts in the initial step of trying to gain permission to enter a foreign market would give us a clearer picture step by step of how Chinese firms are doing in their integration into the world economy.

Foreign market entry attempt failure happens when an original investment proposal is not completed as projected within a given period of time. For example, suppose company A from country B intended to take over company C in country D. Company A proposed a business plan and made contact with company C, but company A or C withdrew from or scrapped the negotiation, and A and C never reached any agreement on this business plan within a given period of time. This attempt would then be called a failed foreign market entry attempt for company A. Various factors could lead to this result. Some could be purely business-related that one or both parties did not meet the requirements/demands of the other so carrying out the business plan was simply not in the interest of one or both parties. This type of failure can often be attributed to the company- and case-specific characteristics. However, there are cases where the demand and supply of both parties are well met but an agreement was never made. For instance, in 2005, China National Offshore Oil Corporation (CNOOC) made a bid to acquire the U.S. energy company Unocal for $18.5 billion. Both CNOOC and Unocal were enthusiastic about this deal: one needed energy and know-how and one needed capital and jobs. But apparently, Washington did not think the same way. The concern of a company with global reach and resources could be under the control of the Chinese Communist Party disturbed many U.S. policymakers, whose objections killed the deal.

Those cases indicate that entering a foreign market is more than a business activity. It was inevitable to be influenced by factors at the macro level of home and host country that are beyond the control of any single firm. It seems to be more so for a country like China which is new to the international arena with comparatively limited experience, “suspicious” ambitions, and an astonishing amount of capital. Certainly, specific characteristics of a firm can make the outcome of a foreign market entry foreseeable to some extent, however, firms have to face the fact that the image they intend to deliver usually is not always in line with the way they are perceived by their partners and host countries. This discrepancy is often caused by the result of differences between the institutional settings of the home and host countries (Zeng and Xu, 2020).

## Conceptual framework

### Firm-level characteristics: Ownership advantage

The characteristics of a company are an important factor in determining an investment’s outcome when discussing OFDI. Favorable characteristics can be called firm advantages. In fact, the study of firm advantage, monopolistic advantage, or ownership advantage has constituted the building blocks of foreign direct investment research since the 1970s (Hymer, 1976). A commonly accepted pattern in IB is that capital expansion usually does not happen until both the economy itself and the actors in it reach a more advanced stage (Dunning, 1973, 1981). Although China has been enjoying rapid expansion for years, its capital outflow is somehow unexpected because China is still at a relatively early stage of development. Therefore, there have been debates about whether Chinese multinationals have firm-specific advantages (Ren et al., 2012). According to the international expansion model that was derived mostly from mature developed country multinationals, firms should have firm-specific assets such as brand, technology, and managerial know-how as their ownership advantages, as they expand to foreign markets (Dunning and Rugman, 1985). The desire to exploit those advantages is believed to be the main driving force for international expansion. It seems that Chinese multinationals do not have those conventionally defined ownership advantages that their western counterparts possessed when they first became involved in IB (Buckley et al., 2007b). Chinese multinationals’ global expansion challenged some of the conventional wisdom. They tend to rely on country-specific assets such as governmental promotion and low labor costs rather than firm-specific ownership advantages. One reason that Chinese multinationals engage in OFDI is to search for and obtain the ownership advantages they lack, which is known as the “springboard” theory (Luo and Tung, 2018).

There is substantial evidence that for many Chinese multinationals, the goal of OFDI is to seek technology and know-how, however, it is not the same as saying Chinese multinationals do not possess ownership advantages *ex ante* (Ramamurti, 2012) as compared to other firms. Moreover, it is hard to explain the variations of Chinese OFDI activities by country-specific assets alone if Chinese multinationals did not have firm-specific ownership advantages. In addition, even if Chinese multinationals’ OFDI were mostly politically oriented in their early stages, they now include firms of various ownership types that have possessed some forms of firm-specific ownership advantages before internationalizing. For example, National Chemical Corporation (ChemChina) had distribution channels and ground organizations before taking over the French manufacturer of animal nutrition additives Adisseo Group in December 2006. Furthermore, Chinese firms have to deal with complex bureaucratic processes in their home country and are thus able to handle similar situations in host countries or regions. Therefore, it is likely that some Chinese multinationals do have ownership advantages but those ownership advantages are acquired using their favorable positions in domestic institutions.

Nonetheless, it is also true that the Chinese firms’ advantages are relatively limited in helping them penetrate foreign markets, especially since sizeable Chinese OFDI only has a short history of roughly three decades and its business models and patterns have yet to be fully formed or recognized by the business player from other countries. Some of the characteristics that are beneficial for firms in the domestic market may not necessarily be seen as advantages when entering an international market. One good example is state ownership. State-owned enterprises (SOEs) are believed to be capable of obtaining support from governments, which can leverage to facilitate their OFDI (Li et al., 2018). However, this enabling view has been contested in some recent studies. For example, Hu and Cui (2014) found a negative effect of state ownership on firms’ internationalization in emerging economies. Huang et al. (2017) also reported similar results among manufacturing SOEs. Profitability may not be the only factor SOEs consider when making internationalization decisions, which may result in undesirable results. Therefore, we hypothesize:

*H1: SOEs are more likely to get in trouble when in a foreign market entry attempt than private firms.*

Unlike their western counterparts, EMNEs often do not possess firm-specific advantages conventionally considered necessary for international expansion as may be inferred from the vastly different internationalization trajectories that some of these firms have pursued (Ramamurti and Singh, 2009). To enhance the likelihood of being recognized as a qualified entity, EMNEs utilize other resources to obtain legitimacy (Rana and Sørensen, 2021). As a way to compensate for their lack of core ownership advantages such as edge-cutting technologies and world-class branding, EMNEs engage in activities that are widely recognized as legitimate. As socially responsible investment has gained prominence, an increasing number of international investors factor firms’ CSR activities into their investment decisions (Scholtens and Sievänen, 2013). According to a KPMG’s 2020 survey,^5^ about 80% of companies worldwide report on their sustainability (compared to 50.1% in 2013 and 20% in 2011). Firms releasing CSR reports following an internationally recognized guideline is usually seen as a commitment to sustainable behavior because those guidelines require self-regulatory and voluntary disclosure of CSR activities (Wagner and Seele, 2017). Firms with CSR reports in accordance with an internationally recognized professional reporting guideline are regarded as legitimate entities. Therefore, we hypothesize:

*H2: Having CSR reports following an internationally recognized guideline is positively related to the probability of a successful market entry attempt.*

In addition, to make up for their lack of managerial resources, some EMNEs hire CEOs with previous overseas experience. It enhances the firm’s capacity of dealing with the complex and uncertain environment in the overseas market (Yi et al., 2021). In addition, CEOs with overseas experience may benefit the firm with their personal networks from overseas markets and improve the firm’s ability to obtain in-depth information and knowledge of the target market (Li, 2018). Thus, we argue:

*H3: Having a CEO with overseas experience is positively related to the probability of a successful market entry attempt*.

### Institutional distance and its moderating effects

The study of the interaction between an organization and its economic and political environment has long been a popular topic in economic sociology and organizational studies (DiMaggio and Powell, 1983, 1991; Zucker, 1987; Stark, 1996; Scott, 2001; Nee and Opper, 2012). Studying organization strategy offers a way to examine the broader institutional circumstances in which economic actors operate and how organizations respond to and interact with them (Fligstein, 1985, 1987; Stark, 1996; Guthrie, 1998). This research analysis is widely used in studies of developing economies (Nee, 1992; Burt, 1995; Walder, 1995; Stark, 1996; Guthrie, 1997). However, this stream of research mainly focuses on organizations’ strategies in the context of operating in domestic markets. For example, the recombinant property strategy employed by large Hungarian enterprises, demonstrated by Stark (1996), is a response to Hungary’s parallel legitimating principles of the decentralized reorganizing of assets and the centralized management of liabilities in Hungary’s property relations restructuring process. In the case of China, Guthrie (Guthrie, 1997) reported that firms adopt a service sector diversification strategy by investing in low-risk and fast-return markets as a way to deal with uncertainty and instability resulting from China’s economic reform. In the same vein, Nee (1992) showed that hybrid forms of organizations were developed among both state-owned enterprises (SOEs) and private-owned enterprises in response to China’s marketization and the changing structure of property rights.

There is an increasing trend in applying institutional theory to the study of IB (Kostova et al., 2008; Peng et al., 2008; Mike et al., 2009; Martin, 2014). Starting with Habib and Zurawicki (2002), who demonstrated the role of an institution on capital flow by considering not only the quality of institutions but also the institutional distance between origin and destination countries, the differences between institutional settings have been widely used in explaining bilateral Foreign Direct Investment (FDI) (Bénassy-Quéré et al., 2007) and firm behaviors (Chao and Kumar, 2010; Salomon and Wu, 2012). Those differences were demonstrated to impact firms’ decisions on location choice (Ramasamy et al., 2012; Lu et al., 2014), entry mode (Agarwal and Ramaswami, 1992; Kwon and Konopa, 1993; Schwens et al., 2011), and strategic choices (Xu and Shenkar, 2002), among others. Since Hymer, one of the founders of IB research, proposed the idea of “LOF”—the cost of doing business abroad that results in disadvantage for multinational companies—in his 1976 book, it has remained an important explanatory variable in the IB field (Werner, 2002; Tsui et al., 2007).

Countries differ from one another on many dimensions. The most commonly used measurement of cross-national distance originated from Hofstede’s (1984) book *Culture’s consequences: International differences in work-related values*, which uses four measurements to capture cultural distance. The concept of cross-national institutional distance was further enriched by scholars such as Ghemawat (2001), who expands Hofstede’s one-dimension measurement of the distance to four-dimension measurements that include cultural distance, administrative and political distance (PD), geographic distance, and economic distance (ED). Despite the rich research employing this concept, there is yet to be a consensus of how to capture the diversity within the concept of institutional distance. One exception is Berry et al. (2010) paper, in which the authors reviewed existing research on cross-national distance and developed a more comprehensive nine-dimension measurement under the framework of institutional theory. They also suggested that researchers should select the dimensions that are most relevant to their subjects. In this study on foreign market entry attempts, we use a three-dimension measurement of institutional distance: PD, ED, and cultural distance.

A political institution is an unavoidable aspect of the national business system. They set rules and regulatory norms to guide and guard business activities. They also constitute an important source of uncertainty for firms as political institutions emphasize the role of the state and they usually constrain firms’ activities in a regulatory way in the interest of the country. The difference between the host and home country may expose foreign firms to political-related hazards, which may have a deterring effect on the firm’s investment activities (Delios and Henisz, 2000, 2003). The difference is correlated with foreign market entry mode and location choice (Garcia-Canal and Guillén, 2008). Economic institution distance refers to the difference between two countries in terms of the factors that determine economic prosperity. It reveals the overall business environment of a country including elements such as macroeconomic environment, infrastructure, and human capital, the measures of which indicate the capability of attracting, absorbing, and conducting foreign direct investment (Whitley, 1992). As the most widely used measurement for cross-national distance, the difference in cultural values and norms has been extensively demonstrated to be an influential factor in foreign market entry and strategy choice (Hofstede, 1980, 1984; Kogut and Singh, 1988).

Greater differences in institutional settings between host and home country indicate a higher risk of being considered “aliens” or in Hymer’s (Hymer, 1976) words, high in “LOF.” Firms from a distant institutional environment often lack credibility and it takes effort to seek acceptance by their host country environment, which is defined as legitimacy (Meyer and Rowan, 1977; Cao and Alon, 2021).

As stated previously, investing in a foreign market is never purely a business activity. It involves a whole range of factors at both macro and micro levels. The impact of country-level variables on the firm-level variable is one of the typically used cross-level interactions in the IB field (Andersson et al., 2014). To what extent a firm’s advantages could guarantee meeting its goal is contingent on the arrangement of the institutional settings of the host and home country. Although distinct institutional settings would lead to variance in behavior outcomes, the distance research suffers from inadequate attention paid to “exactly what mechanisms are at play in the influence of distance” (Zaheer et al., 2012). Therefore, exploring how institutional distance interacts with firm characteristics could supplement current research by providing a more comprehensive understanding of how these two critical mechanisms determine IB practice (Kostova et al., 2020). Some characteristics of a firm might not be an advantage *per se* but could help overcome big institutional gaps. Some other characteristics could be more beneficial to a firm under certain circumstances. It could also be that an advantage may not be as rewarding as expected when a firm faces certain institutional differences.

Treating institutional distance as “liability” is pervasive in IB studies. Barriers, difficulties, costs, and risks are associated with doing business abroad (Stevens and Shenkar, 2012). In the past decade, some scholars have noticed that research in IB may have overly emphasized the negative aspect of distance (Brannen, 2004; Zaheer et al., 2012). A special issue of the *Journal of* IB *Studies*^6^ called for research that views commonly considered phenomena in new ways to develop a rigorous and more complete understanding of how distance works. National distance may mean opportunities for some firms to utilize their own firm characteristics, even if such characteristics are not usually considered an “advantage.” Institutional distance may moderate the relationship between firm advantage and foreign market entry attempt in the way that a favorable position in the home country’s institutional system empowers a firm by providing accessible resources or favorable conditions, which in turn promote a firm’s negotiation power in entering a foreign market (Liang et al., 2014). This favorable position is reflected in a firm’s existing characteristics which they acquired through their interaction with their environment (Porter, 2008). Firms’ characteristics could be at the firm level, carried out by the firm as an entity (such as extant experiences), and some characteristics are directly related to the individuals, especially the top management team (TMT). A line of research has shown that CEOs are reflections of their companies and their backgrounds drive the decisions they make (Hambrick and Mason, 1984; Buyl et al., 2011). Whichever the level is, the functions of those characteristics can hardly isolate from the macro circumstances. Thus, we argue:

*H4: Institutional distance has a moderating effect on the relationship between firm characteristics (company level and individual level) and the likelihood of successfully entering a foreign market.*

## Data and method

A unique dataset is constructed based on the China Global Investment Tracker provided by the Heritage Foundation, which records China’s overseas investments of $100 million or more. The Heritage Foundation is known as a conservative think tank with a strong emphasis on issues of national security and defense. As one of the world’s most influential public policy research institutes, it has influenced several American domestic and foreign policies including the Health Insurance Mandate and American policy on the European Union. Because of its strong orientation toward policy influence, it keeps track of China’s global investments and publishes reports on their trends. It also makes strong conservative claims that the U.S. should “[c]ontinually state at the highest levels that Chinese enterprises that obey American law are welcome” and the U.S. authorities should “closely monitor the behavior of Chinese state firms that are active in the U.S.” Overall, the Heritage Foundation treats China’s investment activities as a potential threat and it closely monitors the trend of its “enemy.” However, its policy-influence orientation and its national defense emphasis are precisely why the information in this dataset can be considered reliable. Gathering accurate information is the first step in knowing one’s enemy. Table 1 depicts geographic distribution of Chinese OFD between 2018 and 2021.

**TABLE 1:** Chinese OFDI by region between 2018 and 2021 (Heritage Foundation).

Region	Investment (in USD)
Asia	91,100
Arab Middle East and North Africa	17,910
North America	29,300
South America	43,350
Europe	107,690
Austrilia	11,540
Sub-Saharan Africa	23,400
Sum	324,290

One of the advantages of using this dataset is that it not only archives transactions that are successful, meaning capital or physical assets that have made their way from China to the host country, but it also includes failed and troubled attempts, meaning one or both parties attempted to reach an agreement but for various reasons, these attempts did not lead to an official signing of the agreement nor its actual execution. Except for several case studies, current quantitative studies in Chinese OFDI have included only successful transactions (Broadman and Sun, 1997; Boateng et al., 2008; Cheung and Qian, 2009; Cui and Jiang, 2012; Cardoza et al., 2014; Lu et al., 2014) and have ignored attempts that were not successful, which renders their findings inconclusive and vulnerable in their explanatory power. The China investment Tracker dataset is the only known dataset that keeps track of unsuccessful and troubled Chinese overseas investments, and thus presents a good opportunity to fill this gap.

### Dependent variable

The dependent variable is a dichotomous variable with 1 equal to successful transactions and 0 otherwise for Chinese overseas investments of $100 million or more in the period of 2018–2021. A transaction will be coded as 0 if an official agreement/contract was not signed. The main sources to determine if an attempt was successful or not include major international media such as Reuters, The Financial Times, Wall Street Journal, and Chinese financial media like Sina Finance. This was further confirmed by those firms’ annual reports; a successful transaction in such a large amount would be considered a “major event” and should be disclosed in any one firm’s annual reports.

### Independent variables

We hand-collected firms and their CEOs’ information of each firm-transaction-year observation from companies’ annual reports and websites. We employ four variables of each transaction to indicate the firm characteristics that are related to foreign direct investment in 2009 and 2010, respectively. Two variables are at the company level and two at the individual (CEO) level.

#### Firm level

A company’s previous overseas experience serves as a source of knowledge about and understanding of doing business abroad (Contractor et al., 2016), which may add credibility to the firm (Lin et al., 2009; Liang et al., 2014). Gaining legitimacy has been argued to be the main motivation for Chinese firms to engage in CSR activities (Zhang et al., 2021). The variable company overseas equals 1 if a Chinese company had previous experience in IB by the time a transaction happened and 0 otherwise. Firms’ socially responsible activities are globally encouraged and are symbols of a firm’s commitment to sustainability (Gu et al., 2022). Previous research has shown that firms use CSR as a means to help overcome low home country legitimacy (Hawn(ed.), 2013). Global Reporting Initiatives (GRI) is a non-profit organization that produces one of the world’s most widely used standards for sustainability reporting for voluntary use. Many Chinese firms are now in line with their western counterparts and employ GRI as the standard for their CSR or sustainability reports. Therefore, a CSR GRI variable is labeled 1 if a firm has published a CSR report following the GRI standard the year before the transaction happened and 0 otherwise.

#### Individual level

CEO overseas equals 1 if the then CEO had worked in a company overseas (including a multinational company in China) or had obtained education such as an MBA from an overseas university when the attempt was made, 0 otherwise. This captures the crucial concept of global mindset and its cultural and strategic value for successful corporate globalization (Levy et al., 2007; Liang et al., 2014); CEO education is a categorical variable with 0 equal to College or less, 1 indicating masters, and 2 meaning a PhD.

### Moderating variables

#### Political distance

We use the PD measurement developed by Berry et al. (2010). Using the Mahalanobis method, the authors calculate the PD with the components of policy-making uncertainty, democracy score, size of the state, world trade agreements, and regional trade agreements from various sources including Political Constraint Index Data (POLCON), Freedom House, the World Development Indicator (WDI), and the World Trade Organization (WTO).

#### Economic distance

The measurement of ED is also from Berry et al.’s (2010), paper, in which they use income, inflation, import, and export and use the same Mahalanobis method to get the ED for each pair of countries. These macro factors are important indicators for a country’s economy as they are related to consumer purchasing power, market stability, and openness to external influence (Whitley, 1992, 1999). The authors also provided updated cross-national distance data on their website through the Center for IB Education and Research at the University of Pennsylvania. The measurements for PD and ED in this study are drawn on the Mahalanobis pooled distance for years 2009 and 2010, respectively.

#### Culture distance

Culture distance (CD) is calculated using the method demonstrated by Kogut and Singh (1988), who based their work on Hofstede’s cultural data. Power distance, uncertainty avoidance, individualism vs. collectivism, and masculinity vs. femininity measurements of each country are obtained from The Hofstede Center website.^7^ It has been shown that the more culturally distant the two countries are, the more distinct are their organizational practices (Hofstede, 1984; Kogut and Singh, 1988; Barkema et al., 1996). A huge gap between a host and home country’s cultural beliefs could potentially result in disparate expectations of companies from those two countries, which in turn could lead to an unsatisfactory outcome.

#### Control variables

Geographic distance is obtained from Google Maps. Investment size is the logged investment amount of each transaction. The sector is a categorical variable following the 4-digit *SIC* codes. The greed field is a binary variable with the value of 1 if a transaction is a green field investment and 0 otherwise. We also include a variable controlling for Belt and Road Initiative (BRI) countries which equals 1 if a country is one of the BRI countries and 0 otherwise. Table 2 provides variable descriptions and measurements.

**TABLE 2:** Variable description, measurement, and sources.

Variable	Description/measurement	Data source
1 Trouble	A binary variable that takes the value of 1 if an investment attempt made by Chinese firms did not lead to the signing of an official agreement/contract within 3 years after the initial negotiation started.	Herigage foundation
2 SOE	A binary variable that takes the value of one if a firm is ultimately controlled by the Chinese government and zero otherwise.	Wind
3 CSR GRI	A binary variable that takes the value of one if a firm publishes a Corporate Social Responsibility (CSR) report following the guidance of Global Reporting Initiative (GRI).	Rankins CSR ratings
4 CEO overseas	A binary variable that takes the value of one if a firm’s CEO has overseas experience (including overseas education and experience working at a foreign firm) and zero otherwise.	Wind/CSMAR
5 PD	Political distance between China and the host country.	Berry et al., 2010
6 ED	Economic distance between China and the host country.	Berry et al., 2010
7 CD	Cultural distance between China and the host country.	Berry et al., 2010
8 Geographic distance	Geographic distance between China and the host country.	Google map
9 Investment	Log value of the investment	Herigage foundation
10 Sector	Four-digit Standard Industrial Classification (*SIC*) codes.	Herigage foundation
11 Greenfield	A binary variable that takes the value of one if an investment is a greenfield investment and zero otherwise.	Herigage foundation
12 BRI	A binary variable that takes the value of one if the target country is part of the Belt and Road Initiatives.	Ministry of Commerce of the People’s Republic of China

## Results and findings

Descriptive data and correlations are summarized in Table 3. The correlations between independent variables are all below 0.5, suggesting that multicollinearity is not a concern. This is further confirmed by the variance inflation factors (VIFs), as all the VIFs are below 2.5 and the average VIF is 2.66, well below the generally accepted multicollinearity threshold of 10 (Hair et al., 2006). The observations in this study are hierarchically nested. Two firms’ attempts to enter a country may share similar characteristics such as the common motivation of seeking natural resources if the country is rich in natural resource reserves. Because the nature of this study involves cross-level analysis, we use the multilevel mixed-effect model to test the effect of firm-level and country institutional level factors on foreign direct investment attempts. This model has been widely accepted and employed in investigating key IB phenomena (Chan et al., 2006; Peterson et al., 2012). Intraclass correlation coefficients (ICC) are below 0.001 which is considered low enough for organizational research according to Hox (2002) criteria.

**TABLE 3:** Descriptive data and correlations.

Variable	Mean	s.d.	1	2	3	4	5	6	7	8	9	10	11	12
1 Troubled	0.137	0.344	1											
2 SOE	0.456	0.498	0.107*	1										
3 CSR GRI	0.513	0.5	–0.08	0.255***	1									
4 CEO overseas	0.584	0.525	0.081*	0.02	0.090*	1								
5 PD	8.025	6.513	0.009	–0.028	–0.044	0.102*	1							
6 ED	32.151	30.227	–0.025	0.108*	0.090*	–0.01	–0.165***	1						
7 CD	23.921	12.487	–0.090*	–0.048	–0.071	–0.024	–0.094*	–0.229***	1					
8 Geographic distance	7734.864	4213.189	–0.006	0.07	0.064	0.134**	0.139***	0.045	0.088*	1				
9 Investment size	5.865	0.957	0.084*	0.211***	0.051	–0.090*	–0.100*	0.053	–0.006	–0.012	1			
10 Sector	5.5237	3.408	–0.059	–0.083*	0.068	–0.027	0.033	–0.087*	0.067	0.022	–0.042	1		
11 Greenfield	0.335	0.472	–0.153***	0.02	0.065	–0.038	0.023	0.144***	0.005	–0.172***	0.029	–0.129**	1	
12 BRI	0.479	0.499	–0.06	0.224***	0.069	–0.082*	–0.152***	0.201***	0.012	–0.192***	0.102*	–0.137***	0.282***	1

*p < 0.05, ^**^p < 0.01, ^***^p < 0.001.

Model 1 is the baseline model with only the control variables. Model 2 adds the three main factors—state ownership (*SOE*), CSR following GRI guideline (*CSR GRI*), and CEO’s previous overseas experience (*CEOEXP)*. Interaction terms between institutional factors and firm-level characteristics are tested in Models 3–5, respectively.

Table 4 reports the regression results. The country-level random effect is very small for each model at *p* < 0.0001 level, so it is not reported in the results. Model 2 provides support for hypotheses 1 and 2, respectively. Compared to private firms, SOEs have a higher chance of facing troubled transactions when trying to enter a foreign market. As predicted, a firm releasing a CSR report following the GRI guideline the year before the transaction is more likely to have a smooth deal than a firm without a CSR report or a report not in line with GRI requirements. Contrary to what we hypothesized in H3, CEO’s previous overseas experience does not contribute successful market entry attempt. The sign in Model 2 for *CEOEXP* is positive but not statistically significant.

**TABLE 4:** Regression results.

	Model 1	Model 2	Model 3	Model 4	Model 5
Troubled = 1					
SOE		0.682**	1.123**	–0.001	1.677**
		(0.287)	(0.535)	(0.404)	(0.663)
CSR GRI		–0.495*	–1.016*	–0.793**	1.349**
		(0.272)	(0.568)	(0.402)	(0.663)
CEO Overseas		0.217	1.630***	–0.030	–0.559
		(0.282)	(0.629)	(0.391)	(0.588)
Political distance (PD)			0.118*		
			(0.066)		
SOE*PD			–0.060		
			(0.055)		
GRI*PD			0.074		
			(0.063)		
CEO overseas*PD			–0.185***		
			(0.070)		
Economic distance (ED)				–0.025**	
				(0.011)	
SOE*ED				0.025**	
				(0.011)	
CSR GRI*ED				0.007	
				(0.010)	
CEO overseas*ED				0.009	
				(0.009)	
Cultural distance (CD)					0.019
					(0.024)
SOE*CD					–0.059**
					(0.026)
CSR GRI*CD					–0.101***
					(0.031)
CEO oversea*CD					0.023
					(0.041)
Geographic distance	–0.001*	–0.000	–0.000	–0.000*	–0.000
	(0.000)	(0.001)	(0.000)	(0.000)	(0.000)
Investment size	0.268**	0.239*	0.237*	0.247*	0.226
	(0.119)	(0.130)	(0.132)	(0.132)	(0.147)
Sector	–0.073**	–0.059	–0.058	–0.072*	–0.051
	(0.037)	(0.040)	(0.041)	(0.042)	(0.044)
Greenfield	–1.185***	–1.032***	–0.931***	–1.077***	–0.752**
	(0.329)	(0.341)	(0.348)	(0.349)	(0.367)
BRI	–0.22	–0.272	–0.318	–0.359	–0.329
	(0.260)	(0.290)	(0.306)	(0.306)	(0.329)
_cons	–2.416***	–2.513***	–3.459***	–1.672*	–3.052***
	(0.787)	(0.889)	(1.076)	(0.942)	(1.139)
N	484	484	484	484	484

Standard errors in parentheses.

*p < 0.10, ^**^p < 0.05, ^***^p < 0.01.

When we add moderating variables into the model, interesting results show up. In Model 3, the interaction term between CEOs overseas and PD gives a negative and statistically significant result. It indicates that although CEO’s existing overseas experience alone does not contribute to a successful deal, it does help to overcome obstacles when a firm enters a politically different country. In Model 4, among the three firm-level characteristics, the interaction term between SOE and ED shows a positive and significant result. It acknowledges that state ownership is more likely to put a firm in a more difficult situation when the firm faces an economically remote market. Finally, in Model 5, we add the moderating variable of CD. Interestingly, both SOE and CSR GRI show a negative and significant result. State ownership might not be a disadvantage anymore when a firm is dealing with a culturally different country. A firm endorsed by the Chinese government may entail legitimacy under certain circumstances. Similarly, engagement in socially responsible activities following the requirement of an internationally recognized standard implicates goodwill. It may be interpreted positively by partners from a distinct cultural background.

Meaningful results are found in control variables as well. Investment size is positively related to the likelihood of engaging in a troubled transaction. Large investments are notable and oftentimes attract closer scrutiny from various stakeholders, which in turn lead to a higher probability of for those investments to be in difficult situations. Likewise, greenfield investments also involve more stakeholders than non-greenfield investments (Müller, 2007) in the host country, and therefore more likely to encounter difficulties.

## Discussion and conclusion

### Contributing to the broader literature

Using a uniquely constructed dataset that contains both successful and failed/troubled Chinese OFDI cases in their initial attempts to enter a foreign market, we found that firm-specific advantages may not always be beneficial when firms try to enter a new market. The effects are contingent on institutional discrepancies between home and host countries. This study contributes to existing IB and institutional theory literature in two ways. First, previous research has largely focused on firms that have made their way through market boundaries (Kolstad and Wiig, 2012; Liang et al., 2014) and thus are considered “successful” in attempting to enter a foreign market, except for sparse cases studies (Huang and Huang, 2013) and media reports, little is known about those that failed in their attempts to invest in a recipient country in the first place. Using a uniquely constructed dataset, we filled this gap by integrating institutional theory with IB theory to show that the dynamic of firm characteristics and institutional distance influences the probability of being successful in a firm’s initial step of penetrating a market boundary. Those unsuccessful attempt cases provide us with more information about the preparation or qualities requisite for a firm to win itself a better start in an IB plan and the obstacles deterring those efforts. In this first phase of investment, differences in political institutional settings between China and the recipient country are more likely to be the factor that dampens the enthusiasm of Chinese investors than the contrast in economic and cultural situations between China and host countries.

Second, this study provides a holistic view of the dynamic of the globalization process. Existing research has studied firm performance in OFDI activities from both macro and micro perspectives. At the macro level, studies have emphasized the country-level determinants of firms’ location choice (Ramasamy et al., 2012; Lu et al., 2014) and entry mode (Agarwal and Ramaswami, 1992; Canabal and White,<suffix>III</suffix>, 2008; Cui and Jiang, 2009; Wei et al., 2014). At the micro level, firms’ context-specific advantages affect their decisions on globalization (Barney, 1991; Cui and Jiang, 2012). This study integrated the two levels of dynamics. Favorable firm-level characteristics are not always beneficial to firms as their effect is constrained by the larger institutional environment the firm is facing when attempting to invest abroad. Distance in political institutions and cultural institutions between China and the host country is more likely to moderate the influence of firm-level characteristics; some are strengthened while others diminish and even are reversed as distance increases.

### Managerial implications

Firms go through several steps in the process of internationalization. Foreign market entry is the first step to testing their internationalization strategies. There is a growing awareness that the difference between the home country and the host country has a profound impact on firms’ international expansion. Managers of multinational companies are increasingly engaging in the proactive arrangement to overcome LOF (Cao and Alon, 2021). Our study suggests that firms should break down institutional distance and consider which aspect of institutional difference has the most critical impact on their international expansion. For example, when cultural distance is the main factor pertaining to their market entry strategy, obtaining a CSR report following GRI guidelines would help to make a legitimate impression on the relevant stakeholders and to increase the probability of a smooth deal. And for companies entering a country with distinct political institutional settings, the CEO’s overseas experience will be a favorable factor when the distance between home and host country increases.

### Limitation and future research direction

The sample used in this study is transactions with an investment amount of 100 million dollars or more. The high stakes may render firms looking to make those investments under closer scrutiny from the host country’s government, which might lead to discretion. This may make these transactions inherently different from those that involve smaller amounts of money. The sample transactions may have made up a majority of China’s OFDI in terms of amount, but they only represent a small number of firms that are conducting OFDI investments. Other firms may have their own unique firm characteristics and face different barriers when attempting to enter a foreign market. Future research should include firms with lower investment stakes if possible. Moreover, this 100-million-dollar threshold has made SOEs overly represented in this sample (81.2%). Privately owned enterprises now constitute almost half of Chinese firms going abroad, they may have distinctive motivation and logic and follow different patterns without the state ownership effect (Cui and Jiang, 2012). These two limitations cannot be easily overcome by the current dataset even though it is the most comprehensive dataset inclusive of unsuccessful market entry attempts. Future research could employ survey methods or conduct case studies to get a more representative body of firms.

## Data availability statement

Publicly available datasets were analyzed in this study. These data can be found here: Heritage Foundation (https://www.aei.org/china-global-investment-tracker/).

## Author contributions

The author confirms being the sole contributor of this work and has approved it for publication.

## Footnotes

1
https://data.worldbank.org/indicator/NY.GDP.MKTP.CD?locations=CN

2
https://unctad.org/topic/investment/world-investment-report

3
https://www.globaltimes.cn/page/202109/1235451.shtml

4
http://finance.sina.com.cn/china/hgjj/20120211/081311358170.shtml

5 Full text of the survey can be found at: https://assets.kpmg/content/dam/kpmg/xx/pdf/2020/11/the-time-has-come.pdf.

6
http://www.palgrave-journals.com/jibs/cfp_positive_organizational_scolarship.html

7
http://geert-hofstede.com/the-hofstede-centre.html
